# Catastrophic Antiphospholipid Syndrome Following Treated Antineutrophilic Cytoplasmic Antibody (ANCA) Vasculitis: Unusual Case Presentation

**DOI:** 10.7759/cureus.83781

**Published:** 2025-05-09

**Authors:** Ahmad Makeen, Nourah A Alorainan, Samar A Baloush, Raghdah Khadwardi, Muhammad Awais

**Affiliations:** 1 Department of Medicine, Nephrology Section, King Abdulaziz Medical City, Ministry of National Guard, Jeddah, SAU; 2 Department of Medicine, King Abdullah International Medical Research Center, Jeddah, SAU; 3 Department of Medicine, King Abdulaziz Medical City, Ministry of National Guard, Jeddah, SAU

**Keywords:** acute renal injury, anca associated vasculitis, antiphospholipid antibody, catastrophic antiphospholipid syndrome (caps), thrombocytopenia

## Abstract

Antineutrophilic cytoplasmic antibody (ANCA)-associated vasculitis (AAV) is a rare autoimmune disorder that affects small blood vessels, leading to systemic symptoms and kidney damage due to the production of autoantibodies. A limited number of case reports have explored the potential link between AAV and antiphospholipid syndrome (APS). Catastrophic antiphospholipid syndrome (CAPS), a severe variant of APS, is associated with high mortality and poor prognosis. We present a case of AAV that initially achieved complete remission, only to later develop CAPS. The presence of antiphospholipid antibodies at baseline may increase the risk of CAPS and thrombotic events in AAV patients. A high level of suspicion for this association, along with early detection of these antibodies, could facilitate prompt diagnosis and enable early, appropriate treatment, potentially improving the patient's outcome.

## Introduction

The rarity of some autoimmune conditions may not allow for a comprehensive portrayal of their clinical presentation. Some autoimmune diseases share overlapping features, which complicates their diagnosis and management. Examples of such conditions include Antineutrophilic cytoplasmic antibody (ANCA)-associated vasculitis (AAV) and catastrophic antiphospholipid syndrome (CAPS).

AAV is a term used for a group of systemic autoimmune disorders characterized by the presence of circulating ANCA immunoglobulins [1]. AAV pathogenesis is yet to be fully understood. However, most pronounced theories attribute its development to immune dysregulation, genetic or external multifactorial, with subsequent neutrophil overactivation leading to vascular damage. Neutrophil activation leads to ANCA production against specific neutrophilic antigens, adding up to the inflammation of small- to medium-sized blood vessels and endothelial damage [2]. The most common types of AAV usually involve both renal and respiratory systems and are typically classified into three entities: granulomatosis with polyangiitis (GPA), microscopic polyangiitis (MPA), and eosinophilic granulomatosis with polyangiitis (EGPA) [1].

Antiphospholipid syndrome (APS) describes a spectrum of autoimmune diseases characterized by the presence of circulating antiphospholipid antibodies (aPL) that alter the normal coagulation physiology and lead to arterial and venous thrombosis and fetal loss.

A devastating outlier of such a spectrum is CAPS. This variant manifests as fulminant multi-organ failure secondary to systemic intravascular thrombosis and small vessel occlusion. The hallmark diagnostic criteria for CAPS are subdivided into definitive and probable criteria. Entities which include the evidence of thrombosis in three or more organ systems, presence of antiphospholipid antibodies on different occasions, intravascular thrombosis evident by histopathology, and development of manifestations usually within one week. In most cases of CAPS, an identifiable triggering factor, such as an infection, surgical procedure, or certain medications, can be found [3].

The pathogenesis of CAPS is attributed to the presence of aPL. These antibodies interfere with the normal function of phospholipids, an essential component of cellular membranes, including those of platelets, endothelial cells, and immune cells. These antibodies work by cellular stimulation, inhibition of endogenous anticoagulants, impairment of fibrinolysis, and overt complement activation, leading to dysregulation of the coagulation cascade, widespread thrombosis, and microvascular occlusion [3].

Despite having distinct diagnostic criteria, both AAV and CAPS can present with similar clinical features, including constitutional symptoms (e.g., fever, fatigue, polyarthralgia, and myalgia), renal involvement (e.g., proteinuria, hematuria, acute renal injury), pulmonary involvement (e.g., alveolar hemorrhage), neurologic manifestations (e.g., peripheral neuropathies) and skin lesions (e.g., livedo, purpura) [1,3]. Given the widespread systemic involvement of those two conditions, the list of differential diagnoses that could also present with a clinically similar picture is just as extensive. Some of these differentials include sepsis, disseminated intravascular coagulation (DIC), heparin-induced thrombocytopenia (HIT), and thrombotic microangiopathies (TMA) [3].

Because the disruption of the coagulation process is non-site specific, CAPS can manifest across all blood vessels, whether venous, arterial, or capillary beds. Therefore, the primary manifestation of CAPS includes abnormal thrombosis in various sites, ranging from large vessels in the peripheral limbs to delicate regions such as cerebral vasculature and renal glomeruli [3]. Despite having different pathogeneses, AAV can also result in extensive thrombosis. However, that is mainly due to inflammatory infiltration of the vascular walls, with occlusions being one of the complications seen in vascular inflammation. In conclusion, both conditions carry a possibility of developing widespread thrombosis, and their coexistence carries an even higher risk.

It is crucial to differentiate between these two conditions in order to guide appropriate management of immunosuppression versus anticoagulation promptly in AAV and CAPS, respectively [4]. However, this clinical overlap creates many diagnostic challenges, necessitating careful consideration of both entities when encountering patients with those symptoms.

Understanding the potential link between AAV and CAPS is crucial for improving diagnosis, risk stratification, and therapeutic management. In this article, we report a patient who developed renal failure secondary to ANCA vasculitis, which was followed years later by CAPS. We will highlight the relationship between AAV and CAPS with additional exploration of the clinical significance of aPL positivity in ANCA patients.

## Case presentation

The patient is a 52-year-old woman with a medical history of hypothyroidism and hypertension. She initially came to our clinic at age 49, reporting a two-week history of fatigue, shortness of breath with minimal exertion, and reduced appetite. She also experienced episodes of nausea and vomiting during this time. In the two days leading up to her visit, her family observed changes in her mental status, including excessive drowsiness and a lack of focus. The remainder of her systematic review was unremarkable.

On physical examination, the patient was drowsy with a Glasgow Coma Scale (GCS) of 14/15 and a delayed response. Her vital signs were as follows: blood pressure was 132/70 mmHg, heart rate was 82 bpm, respiratory rate was 18 breaths per minute, temperature was 36.7°C, and oxygen saturation was 100% on room air. Chest examination revealed bilateral fine crackles, and there was mild edema in the lower limbs. The rest of the physical examination was unremarkable.

Laboratory results indicated acute kidney injury (AKI), hyponatremia, and mildly elevated liver enzymes. At the time of admission, the patient was initially treated for prerenal AKI and hyponatremia, likely due to severe intravascular volume depletion. However, concern for an intrinsic renal cause arose, as her serum creatinine did not improve significantly, and her AKI was quite severe, with a significant number of red blood cells seen in urine microscopy. Consequently, additional investigations for renal impairment were ordered, including anti-glioblastoma multiforme (GBM), ANCA, and an autoimmune profile (Table 1).

**Table 1:** Laboratory serum result of her first admission. Hb: Hemoglobin, WBC: White blood cell count, PLT: platelets, ANA: Antinuclear antibody, DsDNA: double strand DNA, ANCA: Antineutrophilic cytoplasmic antibody, C3, C4: Complement, Anti GBM: glomerular basement membrane antibody

Parameter	Reference Range	Result before admission	Result during admission
Complete Blood Count			
Hb	11.5-16.5 g/dl	11.0 g/dl	11.5 g/dl
WBC	4-11	6.1	5.3
PLT	150-450	289	166
Basic Metabolic Panel			
Serum Creatinine	50-74 umol/L	55 umol/L	650 umol/L
Urea	1.9-5.7 mmol/L	2.7 mmol/L	19.9 mmol/L
Sodium	135-144 mmol/L	136 mmol/L	129 mmol/L
Potassium	3.5-4.9 mmol/L	3.7 mmol/L	3.8 mmol/L
Chloride	101-111 mmol/L	101 mmol/L	93 mmol/L
Bicarbonate	22-29 mmol/L	26 mmol/L	20 mmol/L
Autoimmune Profile			
ANA	Negative	-	Negative
DsDNA	< 200	-	50 IU/ml
P ANCA (MPO)	< 20	-	46.53
C ANCA (PR3)	< 20	-	8.89
C3	0.9-1.8 g/L	-	0.97 g/L
C4	0.1-0.4 g/L	-	0.19 g/L
Anti GBM	< 20	-	4.9

Her renal ultrasound revealed normal-sized kidneys with normal echogenicity and preserved cortical-medullary differentiation. Serology results for infectious and autoimmune diseases were significant only for a positive P-ANCA (MPO) titer of 46.53. She was started on pulse corticosteroids and underwent a kidney biopsy, which confirmed the diagnosis of crescentic ANCA-associated vasculitis, with non-necrotizing granulomas, mild interstitial fibrosis, and tubular atrophy. As a result, she was treated with cyclophosphamide (CYC) induction and received seven sessions of plasma exchange. She also required hemodialysis three times a week as supportive care. Within two weeks of starting CYC, her ANCA titer became negative.

Unfortunately, her hospital course was complicated by bacteremia, recurrent chest infections, and posterior reversible encephalopathy syndrome (PRES). She also experienced episodes of thrombocytopenia, with her platelet count dropping as low as 30. The hematology team attributed this drop to the effects of medications, after ruling out viral infections, heparin-induced thrombocytopenia (HIT), and hemophagocytic lymphohistiocytosis (HLH). After about a month of hospitalization, she was discharged to continue her CYC induction therapy as an outpatient, along with thrice-weekly in-center hemodialysis.

Five months later, she completed her induction therapy, with improvements in both urine output and serum creatinine levels. Her hemodialysis sessions were discontinued, and she showed partial recovery with a stable serum creatinine of around 130 µmol/L. Her maintenance therapy consisted of mycophenolate mofetil (MMF) and a low-dose steroid regimen. MMF was chosen over azathioprine for maintenance therapy due to the patient's prior episodes of severe thrombocytopenia.

**Figure 1:**
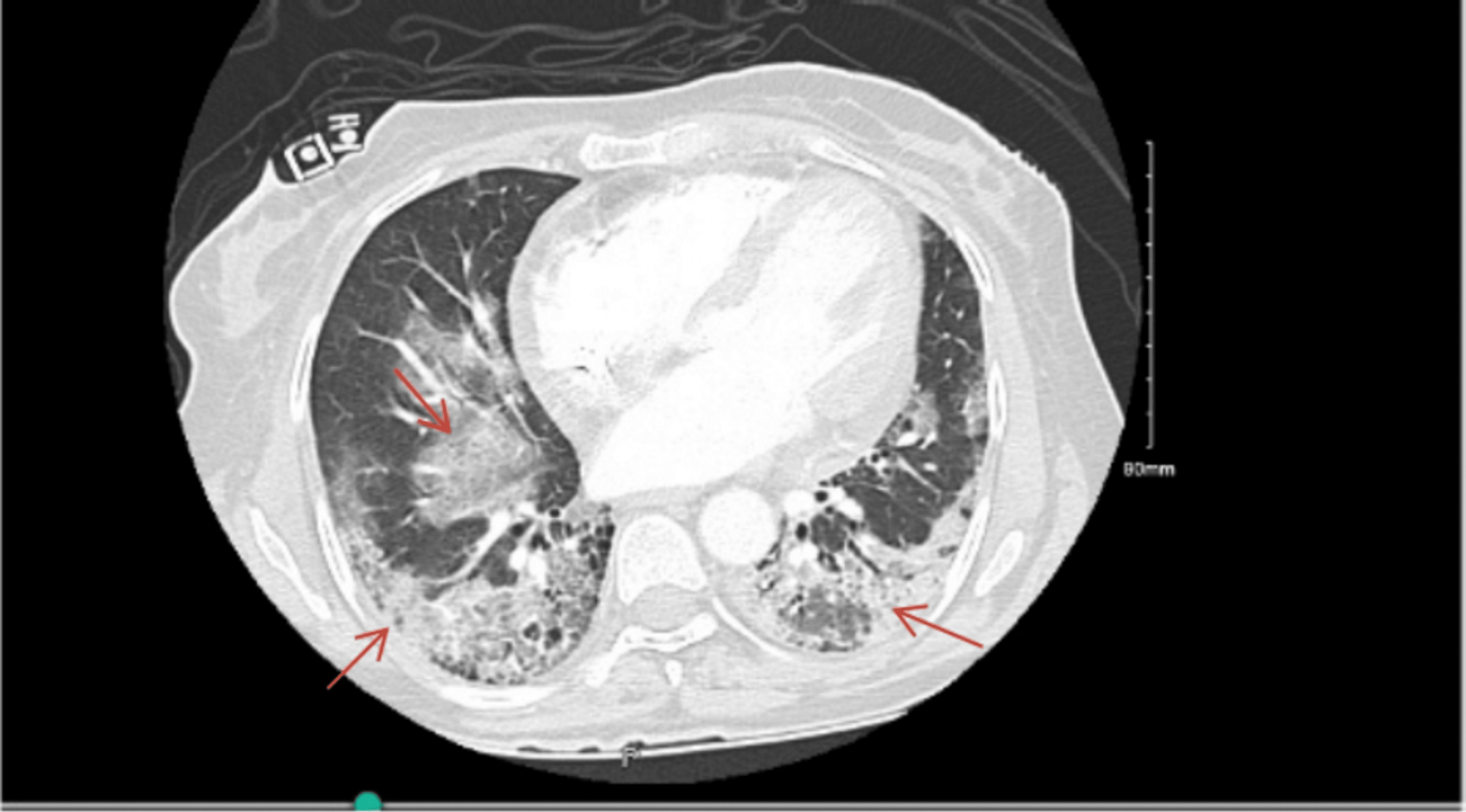
CT chest with contrast. Arrows: Scattered ground glass opacities and honeycombing. Small pericardial effusion noted. No size significant enlarged mediastinal, hilar or axillary lymph nodes sub-centimeter.

Over the next few months, the patient continued follow-up with her nephrologist in the outpatient setting, with no renal complications and persistently negative ANCA titers. However, she had recurrent chest infections and was diagnosed with ANCA-related cryptogenic organizing pneumonia (COP) and fibrotic changes consistent with a non-specific interstitial pneumonia (NSIP) pattern, as seen on a CT scan of the chest arranged by her pulmonologist (Figure 1).

After approximately 30 months of treatment, her mycophenolate mofetil (MMF) therapy was discontinued, and she remained on a low dose of prednisone (5 mg daily). During a subsequent hospitalization for pneumonia, her chronic mild thrombocytopenia worsened. Additional investigations, including antiphospholipid syndrome antibodies, revealed a weakly positive lupus anticoagulant (LA) with the following results: LA1 50 seconds, LA2 36 seconds, and an LA1/LA2 ratio of 1.4, with LA present weekly. A CT chest scan also showed new fibrotic changes, prompting the initiation of antifibrotic therapy for NSIP-related lung fibrosis in the context of ANCA vasculitis. As a result, she was started on Nintedanib 150 mg twice daily, and MMF was resumed. 

Last hospital presentation: three years after initial diagnosis

The patient’s primary complaints were poor appetite, altered mental status (being less alert than usual), and bluish discoloration of her nails. Both the patient and her sitter denied any worsening shortness of breath from baseline, cough, upper respiratory tract infection symptoms, fever, gastrointestinal symptoms, abdominal pain, chest pain, or rashes. On examination, she was found to be in respiratory distress with an oxygen saturation of 69% on room air, hypotension (BP 74/46), tachycardia (HR 106), and a respiratory rate of 24 breaths per minute. Although she was conscious, alert, and oriented, she appeared lethargic. Her blood pressure improved after the administration of 1.5 liters of normal saline, but an attempt at noninvasive ventilation was unsuccessful, and she was intubated. 

Laboratory results showed leukocytosis with a neutrophilic shift, elevated lactic acid, acute kidney injury (AKI), high anion gap metabolic acidosis, and deranged liver and coagulation profiles (Table 2). 

**Table 2:** Laboratory serum result of her last admission. Hb: Hemoglobin, WBC: White blood cell count, PLT: platelets, PT: prothrombin time, PTT: partial thromboplastin time, BGP: beta 2 glycoprotein, LA: lupus anticoagulant, ACA: anticardiolipin antibody, ANCA: Antineutrophilic cytoplasmic antibody, C3, C4: Complement, LDH: lactate dehydrogenase, CK: creatinine kinase, HIT: Heparin induced thrombocytopenia

Parameter	Reference Range	Result before admission	Result during admission
Complete Blood Count			
Hb	11.5-16.5 g/dl	13.2 g/dl	8.9 g/dl
WBC	4-11	4.8	20
PLT	150-450	121	27
Coagulation Profile			
INR	0.8-1.2	1.3	1.9
PT	11-14 sec	14 sec	21 sec
PTT	26-41 sec	37 sec	39 sec
Basic Metabolic Panel			
Serum Creatinine	50-74 umol/L	120 umol/L	275 umol/L
Urea	1.9-5.7 mmol/L	7.2 mmol/L	13.5 mmol/L
Sodium	135-144 mmol/L	138 mmol/L	143 mmol/L
Potassium	3.5-4.9 mmol/L	3.3 mmol/L	3.6 mmol/L
Chloride	101-111 mmol/L	101 mmol/L	111 mmol/L
Bicarbonate	22-29 mmol/L	24 mmol/L	13 mmol/L
Autoimmune Profile			
BGP IgG	<20	-	1.67
BGP IgM	<20	-	1.54
Lupus anticoagulant	Negative	Weakly present	moderately present
LA1	26-44 sec	50 sec	72 sec
LA2	26-38 sec	36 sec	38 sec
LA1/LA2 Ratio	-	1.4	1.9
ACA IgM	<12	6.46	6.98
ACA IgG	<12	2.22	2.77
P ANCA (MPO)	< 20	1.40	3.25
C ANCA (PR3)	< 20	2.26	3.07
C3	0.9 – 1.8 g/L	1.03 g/L	1.03 g/L
C4	0.1 – 0.4 g/L	0.23 g/L	0.25 g/L
Special Biochemical tests			
LDH	100 -217 L/U	478 L/U	784 L/U
CK	27-132 IU/L	483 IU/L	>42670 IU/L
Fibrinogen	2-4 g/L	-	2.4 g/L
Blood Smear	Normal morphology	-	Marked thrombocytopenia, no abnormal cells
HIT	Negative	Negative	Negative

The patient was admitted with a diagnosis of interstitial lung disease (ILD) exacerbation, impending respiratory failure, sepsis, acute kidney injury (AKI), impaired liver function, and thrombocytopenia. She was started on empirical broad-spectrum antibiotics and received 40 mg of intravenous methylprednisolone. Her mycophenolate mofetil (MMF) and nintedanib treatments were temporarily paused. Due to renal impairment, she was initially managed with intravenous fluids and sodium bicarbonate boluses to address acidosis. On reassessment, her hemoglobin dropped from a baseline of 13 to 8.9, and her platelet count decreased from 94 to 27. Further investigations, including a heparin-induced thrombocytopenia (HIT) screen, a DIC workup, and antiphospholipid antibody tests, were sent. She received supportive transfusions.

**Figure 2:**
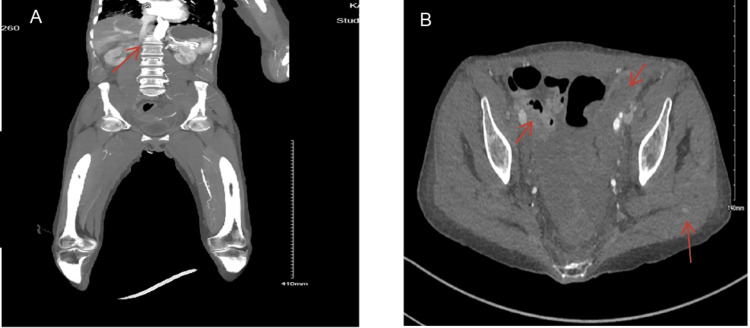
Run off CT with contrast. A: Coronal cut with almost complete occlusion in the abdominal aorta above the level of renal arteries with minimal collateral seen. B: Axial view with the bilateral iliac arteries is faintly opacified with reconstitution via the abdominal wall collaterals at the femoral level bilaterally. There is a faint hyperdensity asymmetric seen at the right glutes muscle keeping with intramuscular active arterial hematoma.

On day three of admission, the patient developed right lower limb mottling and coldness. Examination revealed that the right lower limb, up to the knee, was ice-cold, with noticeable color changes (mottling) and weak, barely palpable pulses bilaterally. Laboratory results that day showed further deterioration, with signs of rhabdomyolysis, including hyperkalemia, hyperphosphatemia, hypocalcemia, raised creatine kinase, and worsening renal function despite initial improvement. Continuous renal replacement therapy (CRRT) was initiated. A contrast-enhanced CT scan was performed, which revealed extensive acute-on-chronic arterial and venous thrombosis extending from the abdominal aorta below the renal arteries to the common iliac vessels, as well as the right superficial femoral artery and its distal branches (Figure 2).

The vascular surgery team evaluated the patient and determined that she had irreversible lower limb ischemia, which was deemed non-salvageable. Given her critical condition, surgical or interventional revascularization was not feasible, with the only potential option being an above-knee amputation (AKA) after stabilization. Unfortunately, anticoagulation therapy was not an option due to her thrombocytopenia and the presence of a psoas muscle arterial hematoma seen on the CT scan. Despite continuous renal replacement therapy (CRRT), her creatine kinase (CK) levels continued to rise disproportionately to the ischemia identified on imaging and clinical examination.

On day seven of her admission, she developed a large area of discoloration on her buttocks and left toe, suggesting more extensive ischemia and myonecrosis, likely due to a higher-level arterial obstruction. The hematology and rheumatology teams were consulted, and the likely diagnosis of catastrophic antiphospholipid syndrome (CAPS) was made, based on her extensive thrombosis and moderately positive lupus anticoagulant. Heparin-induced thrombocytopenia (HIT) was ruled out by negative testing, and there were no signs of hemolysis or schistocytes to suggest disseminated intravascular coagulation (DIC) or thrombotic thrombocytopenic purpura (TTP). Her ANCA test remained persistently negative. Her low platelet count was attributed to high consumption due to thrombosis. She received pulse steroids and intravenous immunoglobulin (IVIG), but there was no significant improvement.

In light of the failure of other therapies, plasmapheresis was attempted, but unfortunately, this did not result in any improvement in her clinical status or platelet count. Given her poor overall condition and grim prognosis, the decision was made to focus on best supportive care without further invasive interventions.

Her condition continued to deteriorate, and she developed sepsis secondary to candidemia, along with pancytopenia. She also experienced gastrointestinal bleeding. At this stage, she was managed with appropriate antimicrobial coverage, conservative measures for the bleeding, and supportive transfusions. Despite all efforts, she passed away after one month in the ICU.

## Discussion

CAPS is a rare and life-threatening manifestation of antiphospholipid syndrome (APS), with an incidence of 0.8% to 1% [5]. Early diagnosis and intervention are critical, as CAPS is associated with a high mortality rate [6-8]. In our case, the clinical presentation met the probable diagnostic criteria for CAPS, as the patient exhibited multi-organ involvement within a week and tested positive for APS antibodies on different occasions [7]. The criteria for diagnosis of CAPS include: multi-organ thrombosis, aPL positivity on repeat testing, histologic or clinical evidence of thrombosis, and acute onset within a week. 

Several differential diagnoses were considered during the patient's management, including thrombotic microangiopathy (TMA), thrombotic thrombocytopenic purpura (TTP)/hemolytic uremic syndrome (HUS), and heparin-induced thrombocytopenia (HIT). These were ruled out based on the absence of hemolysis, a negative HIT screen, and no gastrointestinal manifestations. Sepsis was also present and appropriately managed with broad-spectrum antibiotics. However, as the patient’s condition rapidly progressed over the course of a week, with multiple organ systems affected and laboratory results revealing a positive lupus anticoagulant, CAPS was considered the most likely diagnosis, probably triggered by sepsis. The patient exhibited common CAPS features, including a preceding infection (primarily respiratory), acute respiratory distress syndrome (ARDS), renal failure, and both arterial and venous thrombosis [8,9].

As described in previous reports, CAPS can be triggered in patients with primary APS, systemic lupus erythematosus (SLE), or even present de novo. It has also been associated with other rheumatologic diseases, such as rheumatoid arthritis [8,10], and has been noted as a severe complication in a few cases of systemic sclerosis [11]. However, the association between primary vasculitis and APS is not fully established. In some cases, APS and vasculitis can coexist, but a direct causal relationship between the two conditions has not been definitively proven. One report described a female patient with diffuse alveolar hemorrhage, glomerulonephritis, and positive antiphospholipid antibodies, who was diagnosed with MPO-ANCA vasculitis and APS, and treated with pulse steroids, rituximab, and anticoagulation [12]. Other reports have described cases of concomitant pauci-immune vasculitis and polyarteritis nodosa with APS [13], but to our knowledge, this is the first reported case of CAPS occurring years after the diagnosis of ANCA vasculitis.

Our patient tested positive for lupus anticoagulant antibody, which was part of the investigation into her chronic thrombocytopenia and was confirmed upon repeat testing. She had no previous history of thrombotic or obstetric complications, and other antiphospholipid antibodies remained negative. The presence of lupus anticoagulant in this case may have contributed to the development of CAPS. A retrospective study examining the link between aPL antibodies and ANCA vasculitis found that patients with persistent aPL antibodies were more likely to experience future thrombotic events than those without these antibodies [14]. In a cohort of Japanese ANCA vasculitis patients, 33% tested positive for aPL, and those with positive results had an elevated risk of subsequent thrombotic events [15]. We propose that screening for aPL antibodies in patients with ANCA vasculitis could serve as a useful tool for predicting the likelihood of future thrombosis.

## Conclusions

Previous research indicates a potential link between antiphospholipid syndrome (APS) and small vessel vasculitis. We present a case of catastrophic antiphospholipid syndrome (CAPS) developing years after treatment for ANCA vasculitis. The presence of antiphospholipid (aPL) antibodies in individuals with ANCA vasculitis may increase the risk of future thrombotic events, underscoring the importance of vigilant monitoring for potential complications in these patients.
